# Assessing falls in the elderly population using G-STRIDE foot-mounted inertial sensor

**DOI:** 10.1038/s41598-023-36241-x

**Published:** 2023-06-06

**Authors:** Marta Neira Álvarez, Antonio R. Jiménez Ruiz, Guillermo García-Villamil Neira, Elisabet Huertas-Hoyas, Maria Teresa Espinoza Cerda, Laura Pérez Delgado, Elena Reina Robles, Antonio J. del-Ama, Luisa Ruiz-Ruiz, Sara García-de-Villa, Cristina Rodriguez-Sanchez

**Affiliations:** 1 Department of Geriatrics, Foundation for Research and Biomedical Innovation of the Infanta Sofía Hospital (HUIS), Madrid, 28702 Spain; 2 Spanish National Research Council, Centre for Automation and Robotics (CAR), CSIC-UPM, Arganda del Rey, 28500 Spain; 3 Physical Therapy, Occupational Therapy, Rehabilitation and Physical Medicine Department, Rey Juan Carlos University, Mostoles, 28933 Spain; 4 Geriatrics’s Department, Hospital Universitario de Getafe, Getafe, 28905 Spain; 5 Physiotherapy Department, R. Gascon Baquero, Alcobendas, 28108 Spain; 6 Physiotherapy Department, R. Torrelaguna, Torrelaguna, 28180 Spain; 7 School of Experimental Sciences and Technology, Rey Juan Carlos University, Mostoles, 28933 Spain; 8 Electronics Department, University of Alcalá (UAH), Alcalá de Henares, 28805 Spain

**Keywords:** Health occupations, Engineering, Health care, Geriatrics

## Abstract

Falls are one of the main concerns in the elderly population due to their high prevalence and associated consequences. Guidelines for the management of the elder with falls are comprised of multidimensional assessments, especially gait and balance. Daily clinical practice needs for timely, effortless, and precise tools to assess gait. This work presents the clinical validation of the G-STRIDE system, a 6-axis inertial measurement unit (IMU) with onboard processing algorithms, that allows the calculation of walking-related metrics correlated with clinical markers of fall risk. A cross-sectional case-control study was conducted with 163 participants (falls and non-falls groups). All volunteers were assessed with clinical scales and conducted a 15-min walking test at a self-selected pace while wearing the G-STRIDE. G-STRIDE is a low-cost solution to facilitate the transfer to society and clinical evaluations. It is open hardware and flexible and, thus, has the advantage of providing runtime data processing. Walking descriptors were derived from the device, and a correlation analysis was conducted between walking and clinical variables. G-STRIDE allowed measuring walking parameters in non-restricted walking conditions (e.g. hallway). Walking parameters statistically discriminate between falls and non-falls groups. We found good/excellent estimation accuracy (ICC = 0.885; $p<0.000$) for walking speed, showing good/excellent correlation between gait speed and several clinical variables. G-STRIDE can calculate walking-related metrics that allow for discrimination between falls and non-falls groups, which correlates with clinical indicators of fall risk. A preliminary fall-risk assessment based on the walking parameters was found to improve the Timed Up and Go test in the identification of fallers.

## Introduction

Falls are one of the main geriatric concerns due to their high prevalence, estimated at around 25–28%, and the serious consequences related to falls in the elderly^1^. Approximately one-third of people over 65 and 50% of those over 80 living in the community fall annually, and almost half will fall again in the same year. In the residential setting, these figures are higher, with an estimated 60% of residents falling annually, also associated with a higher prevalence of repeated falls^2^. In addition, falls are an indicator or marker of frailty. Most of the falls in the elderly are associated with physical, functional, and cognitive impairment. Falls also are associated with a higher risk of hospitalization, institutionalization, and loss of quality of life^3,4^.

These factors have led to the publication of guidelines for managing patients with falls. These guidelines highlight the need for a multidimensional assessment, but with emphasis on the assessment of gait and balance^5–7^.

Falls assessment is currently conducted, in both primary and specialized care, by physical examination and functional tests, such as gait speed, Short Physical Performance Test (SPPB), or the Timed Up and Go Test (TUG)^8^. All these tests provide qualitative and quantitative data but are potentially subject to bias due to the subjectivity of the explorer, patient collaboration attitude, and the characteristics of the physical space where the assessments are conducted. Besides, the complete assessment of the patient requires a considerable amount of time, which is limited within daily clinical practice.

With regards to gait assessment, optical systems with cameras (e.g., Vicon or OptiTrack) or pressure sensor floors/walks (e.g., Gaitrite) and posturography, provide accurate information on gait patterns, speed, length, width, and static and dynamic balance behavior under controlled conditions. However, the use of these tools is restricted to specialized clinics with gait/biomechanic laboratories, due to the high cost and specialization needed to exploit this type of technologies, as well as the time and space required to conduct the assessments^9^. In addition, there are some limitations associated with these technologies: firstly, the assessments are conducted in a laboratory environment, not within the patient’s usual environment, where falls usually occur; secondly, conducting the tests requires patient collaboration, which can be complicated in patients with cognitive and/or severe hearing impairment. However, the functional tests currently conducted provide data that cannot be enough to evaluate specific aspects of the gait pattern, or when evaluating improvements following a rehabilitation intervention.

All these reasons led the scientific community to explore the possibility of using cheaper, timeless, easy-to-use, accurate, and automated alternatives for walking assessment. In this context, inertial measuring units (IMU) emerged as an approach of interest for human movement measuring^10–12^.They are lightweight, portable, and low-cost sensors that measure gross accelerations and rotational velocities using triads of orthogonally oriented accelerometers and gyroscopes. The use of IMUs allows gait analysis in real-life conditions, without the need to be in an indoor space or in a hospital. IMUs are placed at the body segments, such as the leg, back, ankle, and foot, measuring accelerations and rotational velocities of the segments. These raw data are further processed to estimate walking parameters with reasonable accuracy. The state of the art of application of inertial sensors for walking assessment has been reviewed in the literature^13^. This review identifies some existing limitations in the literature, most of the studies only include a set of gait parameters and discrepancies are also found among them. Some authors identify certain parameters as significant in identifying frailty or fall risk, while others do not consider them relevant. For this reason, it is essential to develop a device that incorporates all possible parameters.

This background led us to develop an IMU-based prototype for the assessment of the walking characteristics of the elderly, called the G-STRIDE^14^. The objective was to develop a tool that allows us to measure walking parameters precisely, objectively, quickly, and at a low cost (with an estimated hardware cost of 130 € per sensor). In addition, the device would allow assessment outside the clinical or laboratory setting, hence in the natural environment of the patient, and also facilitate measurement in subjects with cognitive impairment. We presented the design of G-STRIDE and the data from a pilot study in which we obtained significant correlations between the walking parameters obtained by G-STRIDE and the commonly used functional tests. However, the reduced sample size of the study did not allow us to derive statistically significant conclusions.

In previous studies^15^, the accuracy of stride length was analyzed, obtaining a mean relative error under 3% for different walking modes. Subsequent studies to test the accuracy, and robustness under a wide range of walking conditions, have been carried out using a high-precision optical system (Optitrack) whose results will be published soon. We can already declare that the found accuracy is quite good with relative errors between 2 and 6% in most gait parameters, and about 30% in clearance. Therefore, the reliability of the estimated gait parameters in the current work can be considered of a similar range.

This article presents the results of a clinical validation study of the G-STRIDE system with proper statistical power and equivalent case and control sample populations. Besides, we also present several improvements in the G-STRIDE device, both in the usability and the algorithms, resulting from the pilot study. Therefore, the main objectives of this article are: to confirm the results from our pilot study, by describing differences between fallers and non-fallers obtained with G-STRIDE, and to investigate the correlations between clinical variables and G-STRIDE walking-related variables.

## Methods

We designed an observational case–control study to investigate whether G-STRIDE provides walking-related metrics that correlate with clinical indicators of fall risk. The study was approved by the Research Ethics Committee of the Hospital Universitario de la Paz (Registration Number: PI-4486). All participants gave informed consent to participate in the study.

The number of subjects to be recruited was calculated from the estimated effect size for a t-test for differences between two independent means based, using data from our previous study^14^ and a statistical power of 0.8 and an alpha error of 0.05. A sample size of 84 subjects per group was obtained.

The complete patient’s dataset, as well as the software used for their gait analysis, is published in G-STRIDE open database at Zenodo^16^.

### Participants

The inclusion criteria for this study are a modification of the criteria for referral to specific units for the evaluation and treatment of falls in the elderly, proposed by the American Geriatrics Society (AGS) and the British Geriatrics Society (BGS)^5^:

- 70 or more years old.
- Able to ambulate without assisting devices, one or two canes.
- Experienced any of the following events:
  - Had a fall with consequences in the last year (requiring medical attention).
  - Had two or more falls.
  - Gait and balance disorder.
  - Fear of falling.
  - Post-fall syndrome.

The exclusion criteria are:

- Having a terminal illness with a life expectancy of fewer than six months.
- Not giving informed consent to participate.

### Assessment tests

Informed consent was obtained from all the subjects participating in this study. The participants were assessed in a single visit to the clinician, followed by the placement of the G-STRIDE device in the patient’s shoe. Then the participant was asked to walk freely outside the office for a minimum of 15 min. The variables collected for this study were the following:

1. Sociodemographic parameters: age, sex, place of residence.
2. Clinical, functional and cognitive variables:
3. Level of physical activity determined accordingly the frailty criteria: Sedentary if patients have less than 3 h of walking per week or activity-related energy consumption lower than 459.6 Kcal in the case of men, less than 1 h or energy consumption lower than 135 Kcal in the case of women.
4. Weight, height, Body mass index (BMI).
5. Degree of cognitive impairment as measured by Reisberg GDS scale (GDS)^17^
6. Frailty assessment using the Standardized Frailty Criteria (FRG)^18^
7. Functional assessment of walking, obtaining the following variables:
  Walking speed (Speed_4m_walk): obtained during a 4-m walking test using the assistive devices she/he normally uses. The actual value is the maximum walking velocity obtained from three consecutive walking tests. A walking speed lower than 0.8 m/s is associated with risk of experiencing adverse events, and below 0.6 m/s with a risk of disability^19^.
  Short Physical Performance Battery Scale (SPPB). This test is specifically designed to predict disability. It has demonstrated the ability to predict adverse events, dependence, institutionalization, and mortality. It is comprised by three categories: (1) balance, evaluated in three positions: feet together, semi-tandem, and tandem; (2) walking speed over 4 m; (3) standing up and sitting down from a chair five consecutive times without using the arms. The subject is considered: autonomous, if he/she obtains 10 or more points; with mild limitation, between 7 and 9; with moderate limitation, between 4 and 6; with severe limitation, less than 4 points^20^.
  Timed Up-and-Go test (TUG): the subject is asked to stand up from a chair without a backrest, walk a distance of 4 m, turn around, return back to the chair and sit down. Completing this test in more than 15 s is associated with high risk of falls^21^.
8. Fear of Falling Syndrome. It is assessed by the Short Falls Efficacy Scale-International (Short FES-I). This is the short version of the 7-item self-administered scale designed to assess fear of falling in older adults living primarily in the community. The 7 items include a variety of functional activities of daily living. The person is asked to score 1 if they are not worried about falling to 4 if they are very worried during different activities. The score ranges from a minimum of 7 (no concern) to 28 (severe concern). From 9 points upwards is considered moderate worry^22^.

We annotate in the logical variable *FALLS* if the participant have had experienced falls accordingly to the criteria proposed by the American Geriatrics Society (AGS) and the British Geriatrics Society (BGS). This data is very important since our main objective is to find differences between participants with and without falls.

### Gait analysis

#### The G-STRIDE system

**Figure 1:**
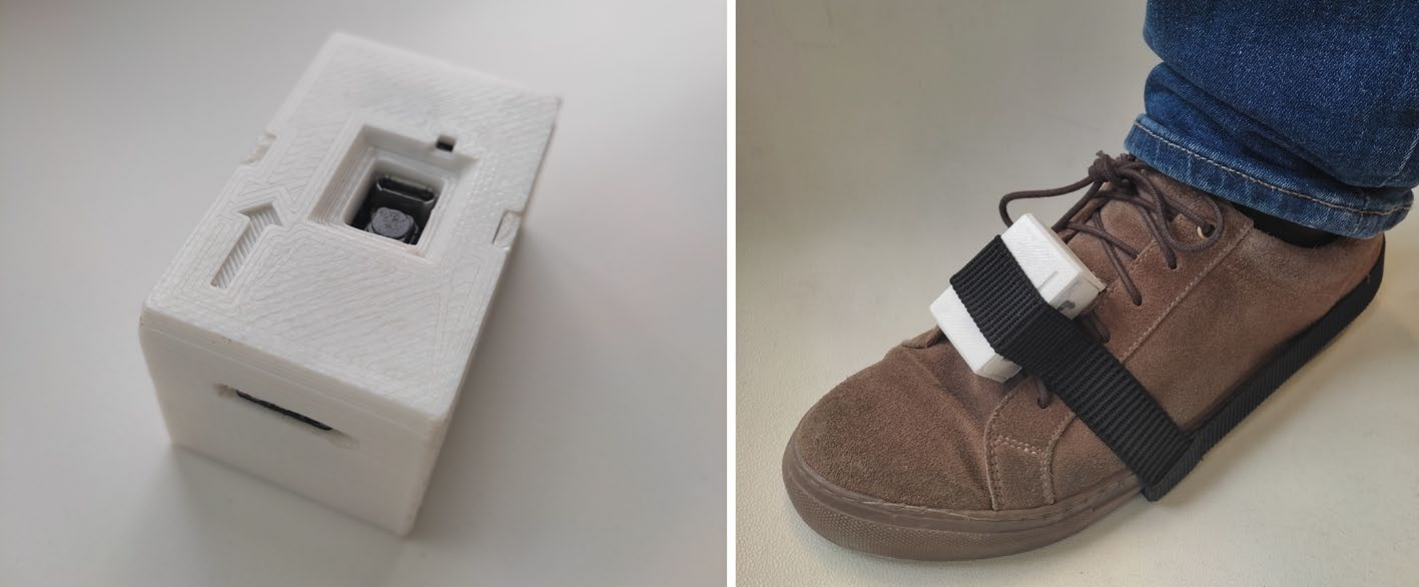
G-STRIDE IMU. Left: unit for tests. Right: IMU attached to a participant’s foot.

The G-STRIDE is comprised by an IMU and Arduino board that samples the data from the IMU during walking. It also features a Secure-Digital (SD) memory card to store the data from each test conducted, as well as Wi-Fi capacity to measure and visualize in real time walking data and system status (Fig. 1). Besides, a Raspberry card is implemented to allow for off-line sensor data analysis stored in the SD card, and the execution of the inertial navigation zero-velocity-update (INS-ZUPT) algorithms to obtain the trajectory and orientation of the foot, and derive a all the walking-related variables defined by the clinicians to assess walking. These variables are then stored in a database hosted in the Raspberry itself and are post-processed in order to obtain the mean and standard deviation (STD) values representing the average walking parameters assessed by the clinicians.

The device is lightweight with dimensions $78 \times 45\times 38$ mm. Noteworthy, no subject had complications or problems derived from the use of the device, and no malfunctions or data loss were experienced. Additional information about INS-ZUPT algorithms can be found in the supplementary material.

#### Estimated gait parameters

The variables obtained by the G-STRIDE are:

- “Total distance (m)”: The total walking distance traveled during every trial, measured in meters.
- “Total time (s)”: The total walking time of every trial, measured in seconds.
- “Total steps”: The total number of steps of every trial.
- “Gait cycle time—GCT”: The mean gait cycle time (GCT), measured in seconds.
- “Velocity (m/s)”: The mean walking speed computed over the total detected steps, measured in meters per second.
- “Cadence (steps/min)”: The mean cadence, as number of steps per minute.
- The time of each cycle in percentage (%) with respect to GCT:
  - “Swing time (% GCT)”: Defined from toe-off to heel strike) as a percentage of GCT.
  - “Stance-loading time (% GCT)”: Defined from heel-strike to start of foot-flat time as a percentage of GCT.
  - “Stance-FootFlat time (% GCT)”: Defined as the time in Foot-flat as a percentage of GCT.
  - “Stance-pushing time (% GCT)”: Defined from the end of foot-flat to toe-off time as a percentage of GCT.
- Pitch angles at start and end of stance/swing:
  - “Heel strike angle (deg)”: The maximum pitch angle at heel strike measured in degrees.
  - “Toe-off angle (deg)”: The maximum pitch angle at toe-off measured in degrees.
- “Stride Length-SL (m)”: The Stride length (distance from one stance position to the next stance of the same foot) measured in meters.
- “StepSpeed (m/s)”: The forward speed of the foot only during the swing phase measured in meters per second.
- “2D Path (m)”: The path length of the foot in the horizontal plane during a step (always equal or larger than SL).
- “3D Path (m)”: The path length of the foot in 3D space during a step (always equal or larger than SL, and 2D path).
- “Clearance (m)”: The clearance or maximum height of the foot with respect to ground during the swing phase.

Additionally to the above-mentioned parameters, which are computed as the mean on a step-by-step basis, we also estimate the standard deviation (STD) among steps. These STD variables are also important to register the regularity or repetitiveness of walk, with higher STD indicating that the gait pattern is not too stable. More information about gait parameters estimation can be found in the supplementary material.

### Database preprocessing

Before the statistical data analysis, we explored and preprocessed the raw data obtained from the device as follows:

- Selecting the variables of interest: we select the relevant variables for the statistical analysis (conventional and IMU-related variables), excluding those variables that do not provide any valuable information for the study (e.g., day and hour of test, identification code of patient, the id of sensor used, etc.).
- Filling missing values: in some cases, any of the clinical variables were not available because of the inability to make a certain test. We detected a few cases and filled out the missing data with the median of the valid observations available for each variable.
- Removing outliers: we identified atypical values that could be due to errors in transcription. If these identified values are not physically possible (e.g., an average speed for a walking elder larger than 2 m/s or 7.2 km/h), those atypical values are removed and substituted by the median value of the valid data.
- Filtering the sample to avoid bias: first, we observe if some of the gait-related parameters are equally distributed among the group of interest (the FALLS group, in our case). When unevenly distributed variables of this type are found, the database could be filtered to make it more evenly distributed by eliminating some observations. If it becomes impossible to eliminate bias in the sampling, then the affected variables (e.g., Age or Height) could be modeled in the regression analysis.

### Statistical analysis

We calculated the sample size for this study by estimating the effect size for a Student’s T test with a statistical power of 0.8 and alpha error of 0.05 (G*Power Software, version 3.1.9). We obtained 84 subjects, and added +5% for losses, for a total of 88 subjects. The remaining analysis was conducted with SPSS v.27 (2013 IBM SPSS Corp.) and the R language for statistical computing (R Foundation for Statistical Computing, Vienna, Austria)^23^ together with the RStudio Integrated Development Environment for R (RStudio, Boston, MA)^24^.

The normal distribution of the variables was tested by the Kolgomorov–Smirnov (KS) test. Continuous variables were analyzed using the mean and standard deviation, and the frequency and percentages for categorical variables. Then, parametric tests were used to measure the differences between independent samples (Student’s T test), as well as the correlations (Pearson’s Test) between the different variables; the Chi-Squared test was used to analyze the differences in percentages.

#### Intraclass correlation coefficients (ICC)

We demonstrate the concurrent validity of each measurement approach (G-STRIDE VS commonly used clinical test) based on the intraclass correlation coefficients (ICCs) calculated using a two-way mixed model (consistency type). Specifically, we evaluated the walking speeds estimated from conventional measurement (“Speed_4m_walk”) and those obtained from **G-STRIDE** IMU (“Velocity (m/s)”) for the long walks. Concurrent validity can be considered very good if ICC is greater than 0.90, good if it is between 0.71 and 0.90, moderate between 0.51 and 0.70, mediocre between 0.31 and 0.50, and bad or null if ICC is less than 0.31, all with 95% confidence interval for the ICC estimate.

#### Correlations between variables

Pearson correlation coefficient was calculated for those variables with a normal distribution, and Spearman’s correlation coefficient for the remaining non-Gaussian distributed variables. Correlation coefficients were interpreted as strong correlation if the coefficient was between 0.50 and 1.00, moderate between 0.30 and 0.49, weak between 0.10 and 0.29, and null between 0 and 0.09.

### Identification of fallers

We assess the potential of the gait variables obtained with the IMUs to improve the identification of fallers compared to the classical functional tests. We use the average and the standard deviation of the IMU variables as inputs of a Support Vector Machine (SVM) classifier^25^ and analyze its classification accuracy. We carry out a random cross-validation with 10 test sets and obtain the average accuracy of the classifier in these test data. The accuracy provided by the SVM is compared with the one obtained by thresholding the outcomes of the TUG test, which is used as a baseline because of its widespread use in the assessment of elderly people.

### Ethics approval and consent to participate

The study was conducted according to the guidelines of the Declaration of Helsinki and approved by the Research Ethics Committee of the Hospital Universitario de la Paz (Registration No: PI-4486). Informed consent was obtained from all subjects involved in the study.

## Results

### Database preprocessing

The database was not filtered, i.e., no complete observations (rows) were eliminated for bias removal in age and height. We identified a few empty or no annotated data that were filled out as described above. The variables and number of missing data were the following: Terrain (11), FRG_weight (1), FRG_energy (9), FRG_strength (3), SPPB_equilibrium (1), SPPB_ChairStand_score (1), TUG (16), FES1 (18). Those missing values represent 0.66% of the total data (60 data out of a total of 9128 data)

**Figure 2:**
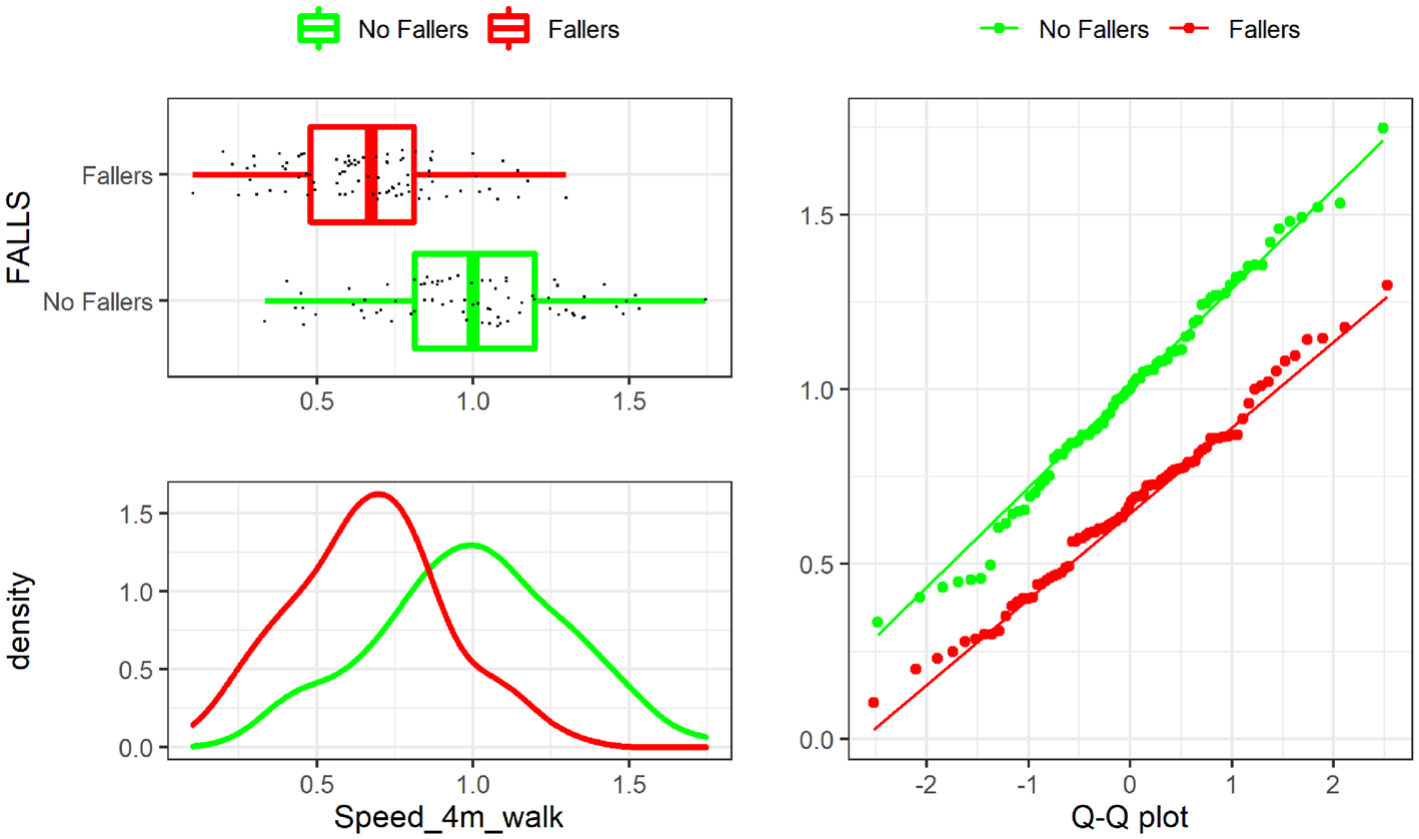
“Speed_4m_walk” normality distribution by Falls group.

**Figure 3:**
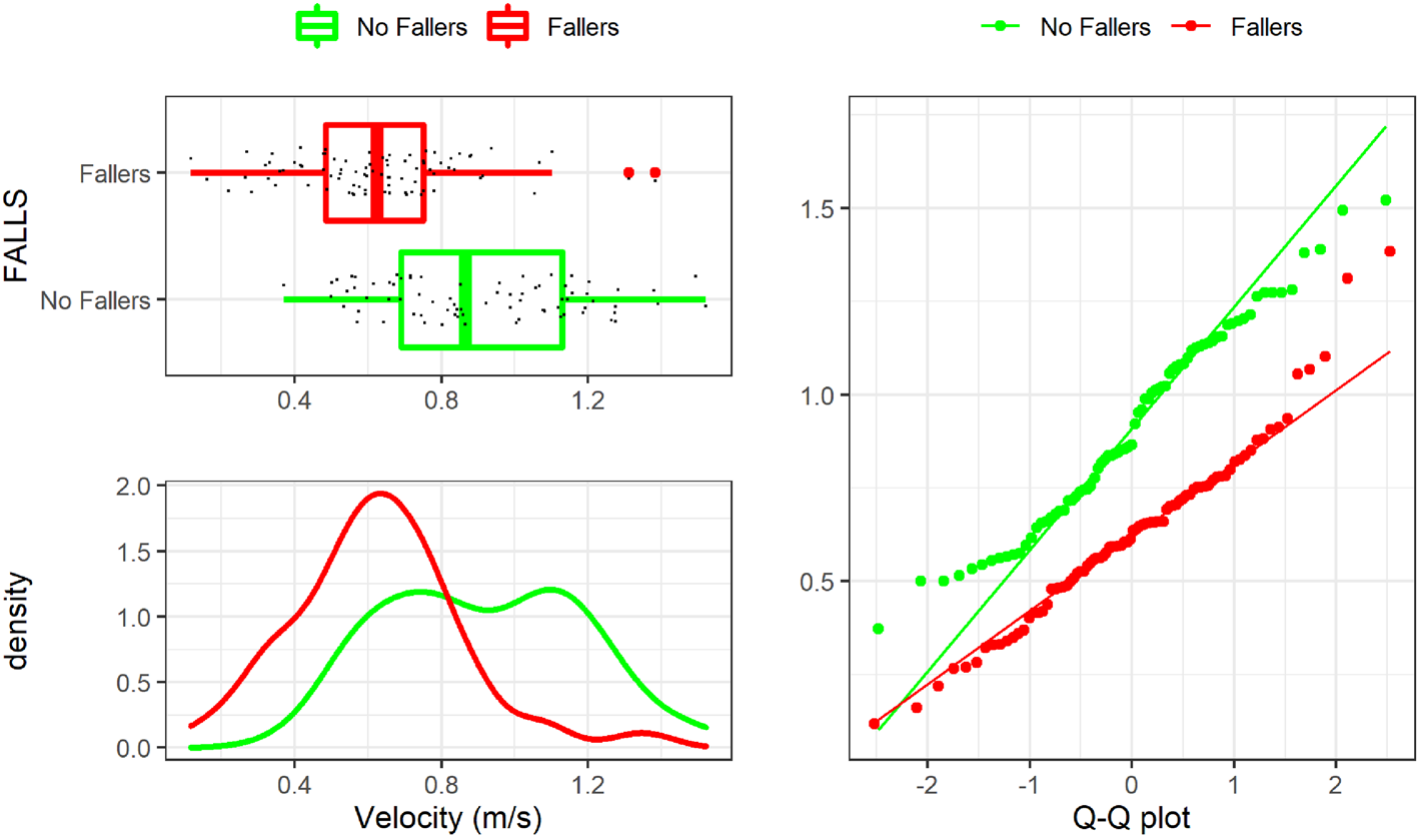
“Velocity (m/s)” normality distribution by Falls group.

The boxplot, a density function, and a Q-Q plot obtained from the KS test for “Speed_4m_walk” is shown in Fig. 2. it It can be concluded that this variable has a Gaussian distribution. Besides, it can also be observed a clear effect size between groups of falls. Only one significant outlier to be removed is detected for this variable (one sample with a velocity larger than 2.0 m/s), and thus it was edited as described above. Similarly, the KS test results for “Velocity (m/s)” are shown in Fig. 3. It can be observed that the distribution of non-falls group is binomial. We hypothesize that either the patients lower their walking speed and get to speeds similar to those in the falls group, or maybe this reduced walking speed is an early symptom of risk of future fall.

### Descriptives statistics

The number of participants that completed the study was 163 subjects: 86 in the falls group and 77 in the non-falls group. The 72% were women (118). The mean age of the overall sample was 82.6 $\pm$6.2 year old, and the mean height was 1.57$\pm$0.1 m. The remaining socio-demographic characteristics by groups of falls, “falls” and “non-falls”, are described in Table 1.

The standard clinical assessment variables are shown in Table 2. A p < 0.001 was obtained for all variables. The null hypothesis about equality of means between groups is rejected in most variables with a significant p < 0.001, except for sex, weight, and Living_site. It is noticeable that the degree of cognitive impairment in the falls group is higher in the falls group as measured by the GDS (p < 0.005).

The walking-related variables obtained from the G-STRIDE are shown in Table 3. We obtained significant differences for all variables (p < 0.001). The descriptives for the inter-step variability are shown in Table 4. We obtained significant differences for GCT-STD, Swing-STD, Stance-Foot-Flat-time-STD, Heel-Strike-Angle-STD, and Clearance-STD (p < 0.001).

In summary, the results show significant differences between the falls and the non-falls groups for all standard clinical assessment variables and the G-STRIDE walking variables.

**Table 1:** Descriptive basal parameters.

Characteristic	Overall, N = 163$^{\text {a}}$	No fallers, N = 77$^{\text {a}}$	Fallers, N = 86$^{\text {a}}$	p value$^{\text {b}}$
Age	82.6 (6.2)/[70.0|98.0]	80.9 (6.5)/[70.0|98.0]	84.2 (5.5)/[71.0|96.0]	$\mathbf{<0.001}$
Sex				0.10
Male	45/163 (28%)	26/77 (34%)	19/86 (22%)	
Female	118/163 (72%)	51/77 (66%)	67/86 (78%)	
Weight	64.3 (13.1)/[33.1|105.0]	65.6 (12.7)/[37.6|105.0]	63.1 (13.4)/[33.1|94.4]	0.4
Height (m)	1.57 (0.10)/[1.32|1.84]	1.62 (0.10)/[1.42|1.84]	1.52 (0.08)/[1.32|1.74]	$\mathbf{<0.001}$
IMC	25.7 (6.1)/[0.0|42.5]	25.0 (4.1)/[16.5|35.5]	26.2 (7.5)/[0.0|42.5]	**0.042**
GDS	2.0 (1.6)/[1.0|7.0]	1.7 (1.4)/[1.0|7.0]	2.3 (1.7)/[1.0|7.0]	**0.004**
Living_Site				0.3
Home	110/163 (67%)	55/77 (71%)	55/86 (64%)	
Residence	53/163 (33%)	22/77 (29%)	31/86 (36%)	
Terrain				$\mathbf{<0.001}$
Flat terrain	110/163 (67%)	33/77 (43%)	77/86 (90%)	
Mix terrain	53/163 (33%)	44/77 (57%)	9/86 (10%)	
Test_Site				$\mathbf{<0.001}$
Hospital	64/163 (39%)	12/77 (16%)	52/86 (60%)	
Residence	52/163 (32%)	22/77 (29%)	30/86 (35%)	
Home	47/163 (29%)	43/77 (56%)	4/86 (4.7%)	

Significant values are in [bold].

$^{\text {a}}$Mean (SD)/[Minimum|Maximum]; n/N (%).

$^{\text {b}}$Wilcoxon rank sum test; Pearson’s Chi-squared test.

**Table 2:** Descriptive conventional parameters.

Characteristic	Overall, N = 163$^{\text {a}}$	No fallers, N = 77$^{\text {a}}$	Fallers, N = 86$^{\text {a}}$	p value$^{\text {b}}$
Time_4m_walk	6.0 (3.9)/[2.0|39.1]	4.5 (1.9)/[2.0|12.0]	7.3 (4.8)/[3.1|39.1]	$\mathbf{<0.001}$
Speed_4m_walk	0.8 (0.3)/[0.1|1.7]	1.0 (0.3)/[0.3|1.7]	0.7 (0.2)/[0.1|1.3]	$\mathbf{<0.001}$
FRG_Total	0.4 (0.8)/[0.0|4.0]	0.1 (0.3)/[0.0|1.0]	0.7 (1.0)/[0.0|4.0]	$\mathbf{<0.001}$
SPPB_Total	8.6 (2.7)/[1.0|12.0]	9.8 (2.4)/[1.0|12.0]	7.5 (2.5)/[3.0|12.0]	$\mathbf{<0.001}$
TUG	15.0 (6.7)/[5.3|36.0]	11.8 (4.7)/[5.3|30.4]	17.8 (6.9)/[7.7|36.0]	$\mathbf{<0.001}$
FES1	10.4 (4.5)/[7.0|32.0]	8.5 (2.7)/[7.0|22.0]	12.2 (5.0)/[7.0|32.0]	$\mathbf{<0.001}$

Significant values are in [bold].

$^{\text {a}}$ Mean (SD)/[Minimum|Maximum].

$^{\text {b}}$ Wilcoxon rank sum test.

**Table 3:** Descriptive G-STRIDE IMU mean parameters.

Characteristic	Overall, N = 163$^{\text {a}}$	No fallers, N = 77$^{\text {a}}$	Fallers, N = 86$^{\text {a}}$	p value$^{\text {b}}$
Total distance (m)	1,006.5 (610.4)/[73.0|2,684.8]	1,321.4 (614.3)/[220.3|2,684.8]	724.5 (449.9)/[73.0|2,636.1]	$\mathbf{<0.001}$
Total time (s)	1,281.6 (424.2)/[338.6|2,184.4]	1,443.5 (397.3)/[338.6|2,184.4]	1,136.6 (396.0)/[369.0|1,921.0]	$\mathbf{<0.001}$
Total steps	1,082.6 (413.6)/[213.0|2,185.0]	1,268.6 (373.8)/[328.0|1,925.0]	916.2 (376.6)/[213.0|2,185.0]	$\mathbf{<0.001}$
Gait cycle time—GCT (s)	1.2 (0.2)/[0.9|1.7]	1.2 (0.1)/[1.0|1.6]	1.3 (0.2)/[0.9|1.7]	$\mathbf{<0.001}$
Stance-pushing time (% GCT)	17.8 (2.7)/[8.1|26.8]	18.6 (2.7)/[12.6|26.8]	17.0 (2.6)/[8.1|24.6]	$\mathbf{<0.001}$
Swing (% GCT)	28.3 (3.5)/[16.1|34.8]	30.0 (2.3)/[23.8|34.1]	26.8 (3.7)/[16.1|34.8]	$\mathbf{<0.001}$
Stance-loading time (% GCT)	11.0 (2.3)/[4.1|16.6]	11.8 (2.3)/[6.5|16.6]	10.2 (2.2)/[4.1|15.4]	$\mathbf{<0.001}$
Stance-FootFlat time (% GCT)	43.0 (7.6)/[26.7|69.8]	39.4 (6.1)/[26.8|53.8]	46.2 (7.5)/[26.7|69.8]	$\mathbf{<0.001}$
Toe off angle (deg)	-52.3 (13.5)/[-84.2|-19.2]	-58.7 (13.2)/[-84.2|-26.3]	-46.7 (11.2)/[-71.7|-19.2]	$\mathbf{<0.001}$
Heel strike angle (deg)	14.8 (7.0)/[0.0|38.0]	17.9 (7.0)/[4.0|38.0]	12.0 (5.6)/[0.0|27.1]	$\mathbf{<0.001}$
Cadence (steps/min)	49.2 (7.5)/[24.0|66.4]	52.1 (5.8)/[38.6|62.9]	46.6 (8.0)/[24.0|66.4]	$\mathbf{<0.001}$
StepSpeed (m/s)	0.8 (0.3)/[0.1|1.6]	1.0 (0.3)/[0.4|1.6]	0.7 (0.2)/[0.1|1.6]	$\mathbf{<0.001}$
StrideLength—SL (m)	0.9 (0.3)/[0.3|1.5]	1.0 (0.2)/[0.5|1.5]	0.8 (0.2)/[0.3|1.4]	$\mathbf{<0.001}$
3D path (m)	1.0 (0.3)/[0.3|1.7]	1.1 (0.3)/[0.5|1.7]	0.9 (0.2)/[0.3|1.4]	$\mathbf{<0.001}$
2D path (m)	0.9 (0.3)/[0.3|1.6]	1.1 (0.3)/[0.5|1.6]	0.8 (0.2)/[0.3|1.4]	$\mathbf{<0.001}$
Clearance (m)	0.16 (0.12)/[0.02|0.55]	0.20 (0.14)/[0.06|0.52]	0.13 (0.09)/[0.02|0.55]	$\mathbf{<0.001}$
Velocity (m/s)	0.8 (0.3)/[0.1|1.5]	0.9 (0.3)/[0.4|1.5]	0.6 (0.2)/[0.1|1.4]	$\mathbf{<0.001}$

Significant values are in [bold].

$^{\text {a}}$ Mean (SD)/[Minimum|Maximum].

$^{\text {b}}$ Wilcoxon rank sum test.

**Table 4:** Descriptive G-STRIDE IMU STD parameters.

Characteristic	Overall, N = 163$^{\text {a}}$	No fallers, N = 77$^{\text {a}}$	Fallers, N = 86$^{\text {a}}$	p value$^{\text {b}}$
GCT STD	0.10 (0.04)/[0.02|0.22]	0.08 (0.04)/[0.03|0.18]	0.12 (0.04)/[0.02|0.22]	$\mathbf{<0.001}$
Stance-pushing time STD	1.87 (0.60)/[0.75|3.68]	1.79 (0.60)/[0.81|3.68]	1.95 (0.59)/[0.75|3.65]	**0.048**
Swing STD	2.38 (0.73)/[0.87|4.54]	2.10 (0.71)/[0.92|4.38]	2.62 (0.66)/[0.87|4.54]	$\mathbf{<0.001}$
Stance-loading time STD	1.68 (0.44)/[0.25|3.17]	1.65 (0.36)/[0.97|2.89]	1.71 (0.49)/[0.25|3.17]	0.2
Stance-FootFlat time STD	4.17 (1.49)/[1.59|9.31]	3.68 (1.46)/[1.63|9.09]	4.60 (1.38)/[1.59|9.31]	$\mathbf{<0.001}$
Toe off angle STD	5.63 (1.40)/[2.74|9.58]	5.61 (1.41)/[2.74|9.58]	5.64 (1.41)/[2.82|8.78]	>0.9
Heel strike angle STD	2.56 (0.87)/[0.01|4.69]	2.84 (0.83)/[0.79|4.69]	2.31 (0.83)/[0.01|4.36]	$\mathbf{<0.001}$
Cadence STD	4.14 (1.29)/[1.31|7.39]	3.79 (1.14)/[1.75|6.88]	4.45 (1.33)/[1.31|7.39]	**0.001**
StepSpeed STD	0.10 (0.03)/[0.03|0.20]	0.11 (0.02)/[0.06|0.17]	0.10 (0.03)/[0.03|0.20]	**0.016**
StrideLength STD	0.11 (0.03)/[0.04|0.21]	0.11 (0.03)/[0.06|0.21]	0.10 (0.03)/[0.04|0.20]	0.051
3D path STD	0.10 (0.03)/[0.04|0.19]	0.10 (0.03)/[0.05|0.19]	0.10 (0.02)/[0.04|0.17]	0.6
2D path STD	0.11 (0.03)/[0.04|0.22]	0.12 (0.04)/[0.05|0.22]	0.11 (0.03)/[0.04|0.20]	0.082
Clearance STD	0.05 (0.03)/[0.01|0.15]	0.06 (0.04)/[0.01|0.15]	0.04 (0.02)/[0.01|0.12]	$\mathbf{<0.001}$

Significant values are in [bold].

$^{\text {a}}$ Mean (SD)/[Minimum|Maximum].

$^{\text {b}}$ Wilcoxon rank sum test.

### ICC results

The Intraclass Correlation Coefficients (ICC) between the “Speed_4m_walk” (measured with a stopwatch) and the “Velocity (m/s)” (measured with G-STRIDE IMU) was 0.885 (F(162,162) = 8.71 , p < 0.0001) within a 95% confidence interval of [0.844–0.916], indicating an accuracy between good and excellent.

### Correlations results

**Figure 4:**
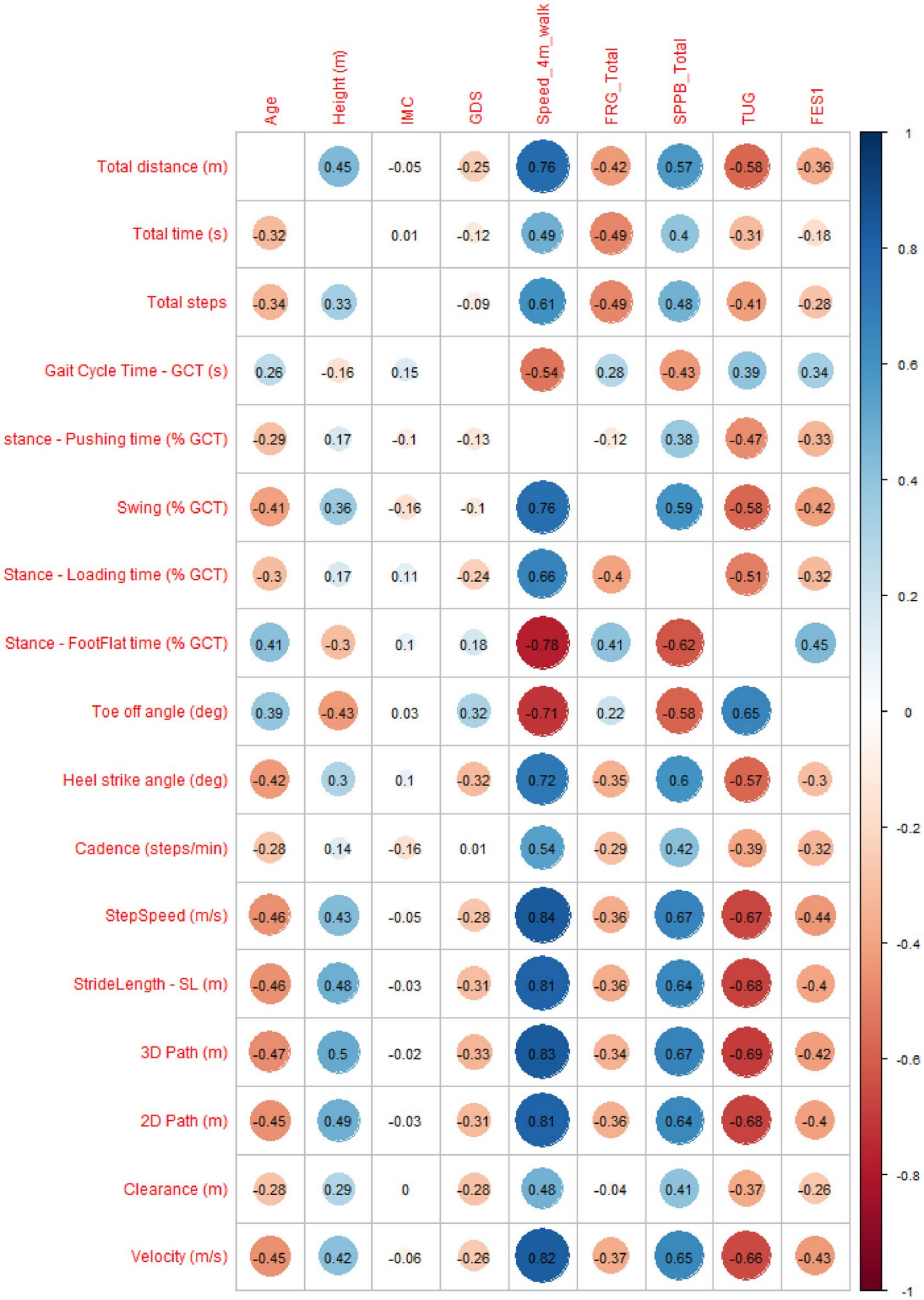
Pearson correlation matrix: Conventional vs IMU parameters. *IMC* BMI or Body Mass Index, *GDS* Global Deterioration Scale de Reisberg, *FRG* Frailty assessment using the Standardized Frailty Criteria, *SPPB* Short Physical Performance Battery Scale, *TUG* Timed Up-and-Go test, *FES1* Falls Efficacy Scale-International.

Correlations between the standard clinical assessment variables and the mean values of the G-STRIDE walking-related variables are shown in Fig. 4. “Speed_4m_walk” is correlated with most G-STRIDE variables, except Stance Pushing Time, Total Time, Cadence, and Foot Clearance.

**Figure 5:**
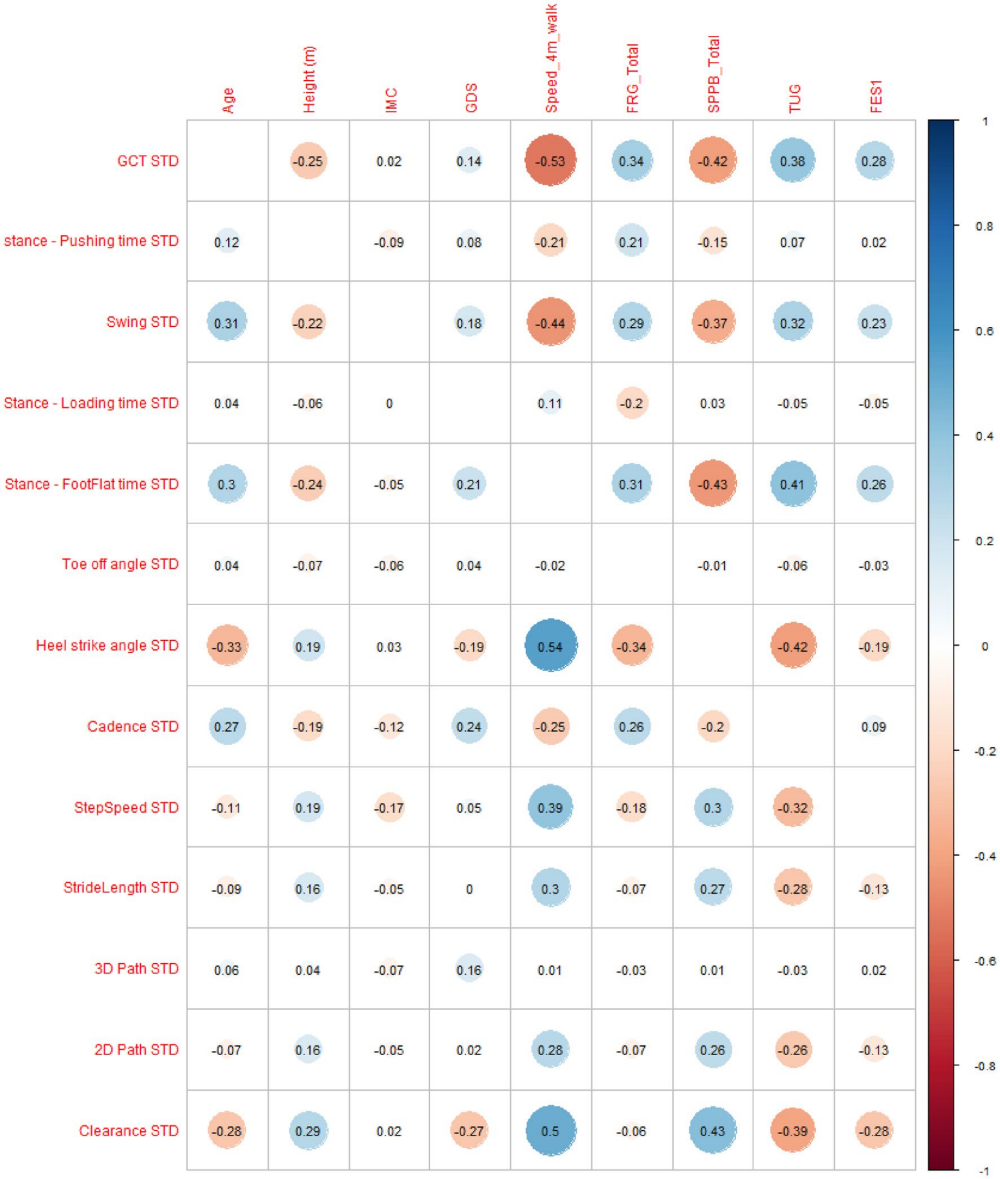
Pearson correlation matrix: Conventional vs IMU_STD parameters. *IMC* BMI or Body Mass Index, *GDS* Global Deterioration Scale de Reisberg, *FRG* Frailty assessment using the Standardized Frailty Criteria, *SPPB* Short Physical Performance Battery Scale, *TUG* Timed Up-and-Go test, *FES1* Falls Efficacy Scale-International.

We also show the correlations between clinical tests and the standard deviation (STD) values of the G-STRIDE walking-related variables is showed in Fig. 5. In this case, “Speed_4m_walk” is partially correlated with GCT STD, Heel Strike Angle STD, and Clearance STD. TUG also correlates with GCT STD and Stance Foot-Flat time STD.

### Identification of fallers

The SVM classifier with the gait variables provides an average accuracy of $73.4\pm 6.0$% for the ten test sets. By thresholding the TUG outcomes, we obtain an average accuracy of $65.9\pm 11.0$% in the evaluation of the same test sets. In this way, the use of the gait variables improves a 11% the classical TUG test for the identification of fallers.

## Discussion

Aging leads to an increase in walking-related problems. The multidimensional evaluation of patients, including the assessment of gait, balance, and physical function of subjects with falls, is part of the recommended practice by clinical guideline^5,6,26,27^. However, these procedures are time-consuming and, in the case of gait analysis, require specialized instruments, highly trained technicians, and specific test conditions (e.g. walkway). Despite the proven usefulness and precision of gait-related data, these factors prevent from being conducted in daily clinical practice. In this context, the aim of this article was to present the clinical evaluation of the G-STRIDE device to assess walking-related variables and indicators of fall risk in the elderly population. Furthermore, we also aimed at describing the differences between the falls and non-falls groups using the variables obtained with the G-STRIDE, and to further study the correlations between these and the standard clinical variables.

The results of this study confirm that the variables obtained from G-STRIDE can discriminate between subjects with and without falls, and show a good-excellent correlation for gait speed (G-STRIDE versus conventional test), as well as a good correlation between most standard clinical assessment variables and those measured by the device.

These results agree with our preliminary results^14^, which showed that the device can accurately measure walking parameters, allowing to discriminate between subjects with and without falls, and with a good correlation between standard clinical variables and those obtained from the device. Furthermore, the device is lightweight, small, and robust, it allows continuous and outdoor real-life analysis, and did not cause any adverse events during use^14^. Although 15-min tests were performed in this study, accurate gait analysis can be obtained in shorter time tests, making it a quick and useful tool. G-STRIDE is a low-cost solution to facilitate the transfer to society and clinical evaluations. It is open hardware and flexible and, thus, has the advantage of providing runtime data processing.

Regarding the baseline characteristics of the sample, we observed significant differences in the age of the subjects, as this is a well-known risk factor for falls: the older the subject, the greater the risk of falls^1,28^. In addition, age is a factor associated with the risk of developing frailty, which, in turn, is closely related to falls^29^. We also found significant differences in the height of the individuals in both groups, with those in the falls group being shorter, which is related to shorter step and stride length, which is also in agreement with findings from other authors^29^.

Another noteworthy aspect is the higher proportion of cognitive impairment among people within the falls group, measured by the GDS assessment scale. This fact is known since cognitive impairment is strongly associated with falls^30^. Attention and executive disorders are closely related to gait impairment, failing one of the most common ones in patients with moderate-severe dementia, but also an early manifestation of mild cognitive impairment^30,31^.

Tests with the device were performed on all types of terrain. However, the falls group tended to walk on flat terrain without obstacles. On the other hand, it is also important to highlight that the evaluations can be performed in all types of settings (residence, home, and clinics), which would allow greater flexibility to the needs of the patient and health system.

Regarding the differences between the two groups, we found that the mean values of the standard clinical assessment variables are different between groups: the participants with falls showed slower gait, longer times in the execution of the TUG, and a greater degree of frailty measured both by standardized frailty criteria or by SPPB, as well as a greater fear of falling. These results are comparable to those observed in the pilot study^14^ and with the results of other authors, which has extended its use as a screening tool in different settings^32–35^.

Similarly, we found significant differences in all the mean G-STRIDE variables, and in the variability of the gait cycle time, swing, stance foot flat time, heel strike angle, cadence, step speed, and clearance variables. The device is capable of assessing up to 17 gait parameters, and this suggests that it may have two main functionalities; first, it allows a broad, objective, and accurate exploration of the entire gait cycle, which is necessary for the evaluation of every patient with falls to rule out gait problems. Furthermore, the preliminary analysis of the identification of fallers based on the G-STRIDE gait variables improved the classification based on functional tests. Secondly, in those older with pathological gait patterns such as Parkinson’s, stroke, or osteoarthrosis, it would allow to establish individualized rehabilitation intervention programs and monitor the response objectively. In this sense, other studies have also demonstrated applicability in specific pathologies such as those mentioned above, finding differences with inertial devices^12,36^.

Finally, the device is easy to use, does not require a specialized setting to measure walking data, and its placement for data collection does not require specialized personnel. Furthermore, the participants reported that the device was comfortable and did not perturb their walking during use, although we did not investigate comfort in depth. These advantages allow the device to be used to continuously monitor daily-living activity, and therefore to derive walking parameters for diagnostic purposes.

We acknowledge the limitations of this study. Firstly, as just explained, we did not measure user (clinicians and patients) acceptance and comfort, being both factors amongst the most relevant barriers to the adoption of technology. Secondly, we did not calculate the costs associated with the use of the device (the device itself and the time consumed by the clinicians to conduct the test), and how this could determine widespread use.

This study ratifies that the G-STRIDE appears to be a suitable tool to measure walking parameters in older adults with falls. It provides precise information on gait pattern, step speed, step length and width, and static and dynamic balance behavior under controlled conditions.

## Conclusions

G-STRIDE allowed measuring walking parameters in non-restricted walking conditions (e.g. a gait walkway), which allows for assessment within the clinic. Gait parameters from G-STRIDE statistically discriminate between the falls and non-falls groups. We found good/excellent estimation accuracy (ICC = 0.885; $p<0.000$) for walking speed, showing good-excellent correlation between gait speed and several standard clinical variables. Finally, in a preliminary classification analysis, we determined that the variables obtained with the G-STRIDE IMU improve a 11% the accuracy in the identification of fallers compared to TUG.

## Supplementary Information

Supplementary Information.

## Data Availability

The data that support the findings of this study are available on zenodo open Access, cited as: García-Villamil-Neira, Guillermo, Neira-Álvarez, Marta, Huertas-Hoyas, Elisabet, Ruiz-Ruiz, Luisa, García-de-Villa, Sara, del-Ama, Antonio J., Rodríguez-Sánchez, María Cristina, Jiménez-Ruiz, Antonio. (2022). GSTRIDE: A database of frailty and functional assessments with inertial gait data from elderly fallers and non-fallers populations (Version v0) [Data set]^16^.
