# Supplementary material for: Assessing falls in the elderly population using G-STRIDE foot-mounted inertial sensor
Source: Sci Rep. 2023 Jun 6;13:9208. doi: 10.1038/s41598-023-36241-x (PMC10244449; doi:10.1038/s41598-023-36241-x)
Supplement: Supplementary file 1 — Supplementary Information. [file 41598_2023_36241_MOESM1_ESM.docx]

**Supplementary materials**

**Assessing falls in the elderly population using G-STRIDE foot-mounted inertial sensor**

**Marta Neira Álvarez^1, *^, Antonio R. Jiménez Ruiz^2, +^, Guillermo García-Villamil Neira^2,+^, Elisabet Huertas-Hoyas^3,+^, María Teresa Espinoza Cerda^4^, Laura Pérez Delgado^5^, Elena Reina Robles^6^, Antonio J. del-Ama^7,+^, Luisa Ruiz-Ruiz^2,8,+^, Sara García-de-Villa ^2,8,+^ and M. Cristina Rodríguez Sanchez**

*marta.neira@salud.madrid.org
^1^Foundation for Research and Biomedical Innovation of the Infanta Sofía Hospital (HUIS), Department of Geriatrics, Madrid, 28702, Spain
^2^Spanish National Research Council, Centre for Automation and Robotics (CAR), Arganda del Rey, 28500, Spain
^3^Rey Juan Carlos University, Physical Therapy, Occupational Therapy, Rehabilitation and Physical Medicine Department, Mostoles, 28933, Spain
^4^Hospital Universitario de Getafe, Geriatrics’s Department, Getafe, 28905, Spain
^5^R. Gascon Vaquero, Physiotherapy Dept, Alcobendas, 28108, Spain
^6^R. Torrelaguna, Physiotherapy Dept, Torrelaguna, 28180, Spain
^7^Rey Juan Carlos University, School of Experimental Sciences and Technology, Mostoles, 28933, Spain

^8^PhD student - University of Alcalá (UAH), Electronics Department, Alcalá de Henares, 28805, Spain

^+^these authors contributed equally to this work

**Gait analysis algorithms**

The algorithms used for gait data analysis are stored in a ZENODO database [1], the software has been developed with Python 3.10.4, and it is available to any user. Download link: <https://doi.org/10.5281/zenodo.6883292>

Sensor signals from accelerometers and gyroscopes are processed to obtain velocity and trajectory. The method used is inertial integration with zero velocity updates (INS-ZUPT) [2, 3]. This algorithm consists of integrating the acceleration to obtain velocity with drift corrections (zero velocity updates) every time the sensor is stationary (i.e., a step is detected previously), and then integrating the velocity to estimate the trajectory. Figure 1 shows a detailed diagram of this algorithm, where the first part corresponds to the step/stance detection phase and the second part corresponds to the INS-ZUPT algorithm.


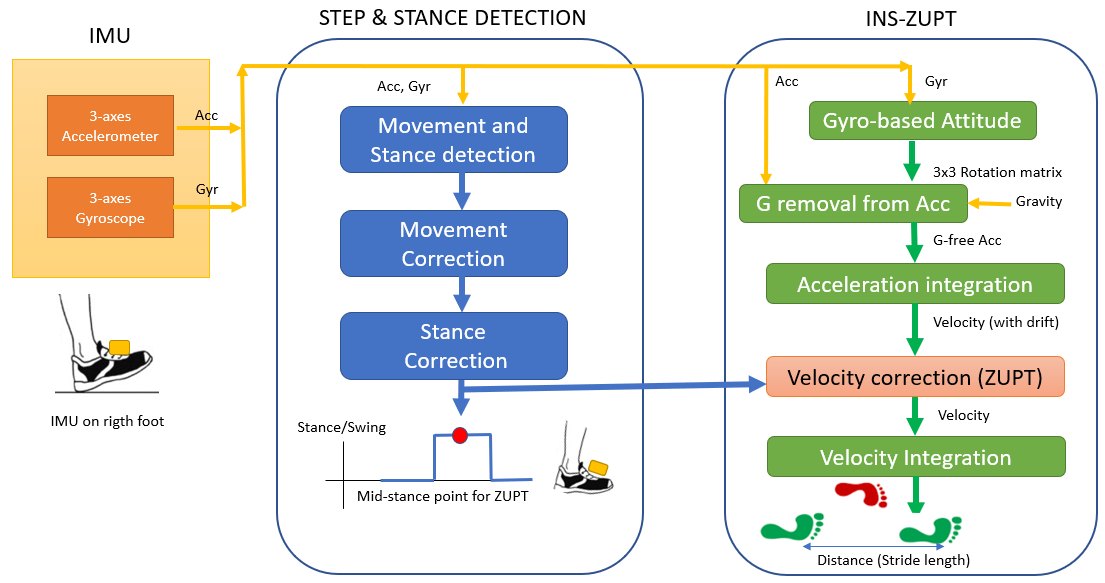


Figure 1. Algorithm diagram including detection phase (blue) and INS-ZUPT phase (green) from [3].

**Gait parameters estimation**

Gait parameter estimation is performed from step segmentation; for this purpose, it is necessary to identify the points of heel-strike (HS), toe-off (TO), and foot-flat (FF). A stride begins with a heel strike (HS_initial) and ends with the next heel strike of the same foot (HS_final).

The gait phases are identified from the foot pitch angle, where HS and TO correspond to the maximum positive and negative peak respectively, while FF phase is obtained from the gyroscope magnitude signal using a threshold < 0.5 rad/s.

From these points, the different parameters are obtained for each step according to the following equations: time is the sensor timestamp, XYZ are the trajectory coordinates, pitch is the sensor pitch angle, and deltas2d or deltas3d are the displacement in XY and XYZ, respectively.

- Gait Cycle Time-GCT = time [HS_initial] – time [HS_final]
- Cadence = 60 / GCT
- Swing time (% GCT) = (time [HS_final] – time [TO]) *100/GCT
- Stance time (%GCT) = (time [TO] – time [HS_initial]) *100/GCT
- Stance-Loading time (%GCT) = (time [FF_initial] – time [HS_initial]) *100/GCT
- Stance-FootFlat time (%GCT) = (time [FF_final] – time [FF_initial]) *100/GCT
- Stance-Pushing time (%GCT) = (time [TO] – time [FF_final]) *100/GCT
- Heel-strike angle (deg) = pitch [HS_initial]
- Toe-off angle (deg) = pitch [TO]
- Stride Length – SL (m) = sqrt ((X [HS_final] – X [HS_initial])^2^ + Y [HS_final] – Y [HS_initial])^2^))
- Step Speed (m/s) = SL/GCT
- 2D Path (m) = sum (deltas2d [HS_initial: HS_final])
- 3D Path (m) = sum (deltas3d [HS_initial: HS_final])
- Clearance (m) = max (Z) – min (Z)

**Absolute and Relative Mean Errors**

The errors obtained in the estimation of the parameters have been calculated using the Optitrack optical system as a reference and the Rizzoli Lower Protocol with 26 markers located in the legs and feet. Evaluation tests have been carried out by 3 subjects, who wore the sensors attached to their feet.

Both systems (inertial and optical) were synchronized at the beginning of each test performing a synchronous movement with the feet. In this way, the steps have been synchronized and the errors for each of them have been calculated. The absolute and relative mean errors obtained for each parameter are shown below in Table 1.

**Table 1. Gait Parameters Mean Relative Error (%) and Mean Absolute Error**

| Parameter (unit) | Relative Error (%) | | Absolute Error (unit) | |
| --- | --- | --- | --- | --- |
|  | Mean | STD | Mean | STD |
| Gait Cycle Time (s) | 2.67 | 2.61 | 0.04 | 0.05 |
| Cadence (steps/min) | 2.67 | 2.61 | 2.73 | 2.66 |
| Stride Length (m) | 2.55 | 2.48 | 0.03 | 0.03 |
| Path Length 2D (% SL) | 1.45 | 1.36 | 0.02 | 0.01 |
| Path Length 3D (%SL) | 2.23 | 1.55 | 0.02 | 0.02 |
| Clearance (m) | 25.18 | 9.12 | 0.03 | 0.01 |
| Velocity (m/s^2) | 2.53 | 1.95 | 0.02 | 0.02 |
| Per Stance(% GCT) | 8.41 | 4.69 | 5.13 | 2.60 |
| Per Swing (% GCT) | 12.32 | 6.56 | 5.13 | 2.60 |
| Per Loading (% Stance) | 40.74 | 18.65 | 8.26 | 3.90 |
| Per FootFlat (% Stance) | 38.26 | 24.48 | 16.98 | 11.27 |
| Per Pushing (% Stance) | 21.15 | 16.82 | 10.77 | 9.97 |

These results are comparable to those reported in the literature, such as in the case of [4], which shows a mean absolute error of 2.52 ± 3.61 cm for clearance and 0.96 ± 1.24 for step length (equivalent to half stride length).

|1] García-Villamil Neira, Guillermo, Neira Álvarez, Marta, Huertas Hoyas, Elisabet, Ruiz Ruiz, Luisa, García-de-Villa, Sara, del-Ama, Antonio J., Rodríguez Sánchez, María Cristina, & Jiménez Ruiz, Antonio. (2022). GSTRIDE: A database of frailty and functional assessments with inertial gait data from elderly fallers and non-fallers populations (Version v0) [Data set]. Zenodo. https://doi.org/10.5281/zenodo.6883292

[2] A. R. Jiménez, F. Seco, C. Prieto, and J. Guevara, A comparison of pedestrian dead-reckoning algorithms using a low-cost MEMS IMU, in WISP 2009 - 6th IEEE International Symposium on Intelligent Signal Processing - Proceedings, 2009, pp. 37–42

[3] García-Villamil, G., Ruiz, L., Jiménez, A.R., Granja, F.S., & Rodríguez-Sánchez, M.C. (2021). Influence of IMU's Measurement Noise on the Accuracy of Stride-Length Estimation for Gait Analysis. IPIN-WiP.

[4] R. Liu et al., "A Wearable Gait Analysis and Recognition Method for Parkinson’s Disease Based on Error State Kalman Filter," in IEEE Journal of Biomedical and Health Informatics, vol. 26, no. 8, pp. 4165-4175, Aug. 2022, doi: 10.1109/JBHI.2022.3174249.
